# Non-consensual and Consensual Non-monogamy in Norway

**DOI:** 10.1080/19317611.2021.1947931

**Published:** 2021-07-12

**Authors:** Bente Træen, Frode Thuen

**Affiliations:** a Department of Psychology, University of Oslo, Oslo, Norway; b Western Norway University of Applied Sciences, Bergen, Norway

**Keywords:** Extradyadic, non-consensual non-monogamy, consensual non-monogamy, condom use, Norway

## Abstract

The paper sets out to study Norwegians’ experiences of non-monogamy. Data were collected by questionnaires in a web sample of 4160 Norwegians (18–89 years). 26.3% of men and 17.8% of women reported that they ever had non-consensual non-monogamy. Consensual non-monogamy was reported by 3%. Compared to participants with no or non-consensual experience, consensual non-monogamy was highly related to relationship intimacy and positive sexual attitudes toward sex and sexuality. At the most recent extradyadic event, 21.5% of heterosexual men and 47.1% of gay/bisexual men reported condom use, which implies a risk for sexually transmitted diseases.

## Introduction

Monogamy in committed relationships is a central norm in most societies and cultures (Blow & Hartnett, 2005a). An important characteristic of committed relationships where monogamy is agreed upon and expected is that sexual interaction is regarded as acceptable only for the two individuals involved in the relationship (Luo et al., 2010). Accordingly, when one of the partners engages in sexual interaction with someone outside the primary relationship without the partner’s consent (subsequently called an extradyadic partner), such behaviors may be termed “non-consensual non-monogamy.” Likewise, it may be termed “consensual non-monogamy” if an individual engages in sexual interaction with someone outside the primary relationship with the partner’s consent. It should be noted that monogamy agreements refer to more than just sexual activity with someone outside a romantic relationship. There are many forms of non-consensual non-monogamy, for instance, romantic, or online.

People of all ages tend to condemn non-consensual non-monogamy as a serious break from social norms (Feldman & Cauffman, 1999; Watkins & Boon, 2016). Nevertheless, a considerable number of people worldwide engage in non-consensual non-monogamy. Bearing in mind that different studies use different definitions of non-monogamy (Blow & Hartnett, 2005a, 2005b), it is estimated that non-consensual non-monogamy occurs in <25% of committed relationships in the USA. According to Luo et al. (2010), the prevalence of non-consensual non-monogamy can far surpass 25% depending on how it is defined. In Norway, 17% of the married or cohabiting population in 1987, 15% in 1992, and 13% in 2002 claimed they had had an extradyadic sexual partner during their current relationship (Træen et al., 2007; Træen & Stigum, 1998). However, in these previous studies, no distinction between consensual and non-consensual non-monogamy was made, and worldwide, few studies have made this distinction. People have different ways of organizing their lives together, and some engage in non-monogamy with their partners’ consent (Barker & Langdridge, 2010; Blow & Hartnett, 2005a; Hackathorn & Ashdown, 2021; Haupert et al., 2017a; Rodrigues et al., 2019; Rossman et al., 2019; Vaillancourt, 2006). North-American studies using convenience samples have estimated that 3–7% of the population is currently in relationships that permit non-monogamy (Haupert et al., 2017a, Rubin et al., 2014). However, it is estimated that a substantially higher proportion, ∼20%, has been part of a consensual non-monogamy relationship at least once during their lifetime (Haupert et al., 2017b).

Studies on non-consensual non-monogamy have focused on the relationship with, e.g. gender, age, sexual orientation, attitudes toward sex and sexuality, emotional closeness to the primary partner, and relationship satisfaction (Allen et al., 2005; Blow & Hartnett, 2005a; Drigotas & Barta, 2001; Hackathorn & Ashdown, 2021; Martins et al., 2016), but less is known about what consensual non-monogamy is associated with. The present paper sets out to explore the prevalence of consensual, and non-consensual non-monogamy in the Norwegian population, and what separates people who are monogamous, consensual, and non-consensual non-monogamy, respectively, with regards to relationship intimacy and attitudes toward sexuality. In addition, we explore the extent to which non-monogamy, whether consensual or non-consensual, represents a risk of contracting sexually transmitted infections (STI). To the latter, we explore the circumstances around non-monogamy, what type of sex people have, and whether or not condoms are used during non-monogamy. Non-monogamy is a field of research with no strong theoretical anchoring, and studies have mainly had a behavioral epidemiology approach when exploring such activity. The present study is also of a behavioral epidemiological approach, and the selection of variables for the analyses is based on previous literature.

### Gender, age, and non-monogamy

A common finding is that more men than women have non-consensual non-monogamy experiences, and men have had more extradyadic partners than women (Atkins et al., 2001; Blow & Hartnett, 2005a; Lewin et al., 2000). Men also seem to be less emotionally involved with their extradyadic partners than women are (Banfield & McCabe, 2001; Buss & Shackelford, 1997; Hackathorn & Ashdown, 2021; Kelly & Byrne, 1992). Studies of non-consensual non-monogamy often emphasize that men have greater sexual interest than women. However, recent studies show that this difference is diminishing (Burdette et al., 2007; Martins et al., 2016). In the past decades, women have changed their sexual behavior, including non-monogamy, more than men have (Blow & Hartnett, 2005a; Lewin et al., 2000; Traeen et al., 2007; Traeen & Stigum, 1998; Valkenburg & Peter, 2007).

Wiederman (1997) found that gender differences in non-consensual non-monogamy in the USA had nearly disappeared among persons below 40 years of age, and discussed whether this is an effect of the so-called sexual revolution. Among other things, the sexual revolution in the 1960s and 70 s meant more liberal sexual attitudes, change in the traditional female gender role, and access to secure female contraception (the pill). Atkins et al. (2001) found that women’s experience of non-monogamy increased up to the age of 40 years and dropped for women above the age of 40 years. Men aged 40–60 years were most likely to report having had an extradyadic partner, and the likelihood decreased significantly among older men. However, as emphasized by Blow and Hartnett (2005b), as most studies are cross-sectional, there is no way to decide if it is a development effect or a cohort effect. Even so, Kontula and Haavio-Mannila (1995) concluded that much of the sexual behavior change in Finland is a generational phenomenon. Social changes across generations may produce different attitudes toward sex and sexuality and norms in various age groups. Today’s younger adults have grown up in a time of greater acceptance of sexual minorities’ rights and emphasis on sexual diversity. Therefore, younger adults are expected to be more likely than older adults to have engaged in consensual non-monogamy. On the other hand, the lifetime experience of non-monogamy, whether consensual or non-consensual, is more likely to be a reflection of the period in which the individual has lived (Træen & Stigum, 1998).

In this study, we hypothesize that men will have more experience of non-consensual non-monogamy than women, but that there are no gender differences in consensual non-monogamy. Furthermore, we hypothesize that the gender differences are less pronounced in younger than in older individuals.

### Sexual orientation and non-monogamy

Sexual orientation is another factor associated with non-monogamy (Blow & Hartnett, 2005a; Blumstein & Schwartz, 1983). Kurdek (1991b) found evidence that individuals who identify as gay and lesbian have more experience with non-monogamy than individuals who identify as heterosexuals. However, as of today, this is not widely studied, most likely because sexual minorities have traditionally faced stigmatization and stereotypes that emphasize sexual promiscuity (Pinsof & Haselton, 2017). However, a qualitative study by Worth et al. (2002) concluded that non-monogamy in some gay relationships may be regarded as more acceptable, and does not cause jealousy and pain. On that background, we hypothesize that individuals who identify as lesbian, gay, bisexual, and transsexual (LGBT+) will have more experience of both non-consensual and consensual non-monogamy than individuals who identify as heterosexuals.

### Attitudes toward sex and sexuality and non-monogamy

Research has shown that people with more permissive attitudes toward non-monogamy are more likely to have engaged in (nonconsensual) non-monogamy (Blow & Hartnett, 2005a; Solstad & Mucic, 1999; Treas & Giesen, 2000). However, the direction of this relationship is not clear. Based on Balzarini et al. (2020) study among swingers, it may be assumed that people who have consensual non-monogamy have more permissive general sexual attitudes and are more unrestricted sexually than monogamous people. Even though individuals who have consensual non-monogamy are not synonymous with swingers, they may also be more sexually permissive than most people are. Accordingly, one can reasonably expect that individuals who give their consent to non-monogamy have more permissive sexual attitudes than the majority of the population.

### Relationship quality and non-monogamy

It is well-documented that important motives for non-consensual non-monogamy are dissatisfaction with the primary relationship (Blow & Hartnett, 2005a; Bui et al., 1996; Christopher & Sprecher, 2000; Drigotas et al., 1999; Hackathorn & Ashdown, 2021; Impett et al., 2001; Kurdek, 1991a; Previti & Amato, 2004; Traeen & Stigum, 1998; Treas & Giesen, 2000). Omarzu et al. (2012) explored the motivations for non-monogamy and emotions connected to it in 22 men and 55 women engaged in extramarital relationships. They found that sexual needs, emotional needs, and falling in love were the most important reasons for non-monogamy. Both genders were equally likely to report sexual or emotional motivations if either of these elements were not satisfactory in their primary relationship. Furthermore, most participants reported experiencing both negative and positive emotions related to non-monogamy. However, much less is known about the motivation for those who engage in consensual non-monogamy. The few studies that exist indicate that those who engage in consensual non-monogamy tend to have a higher socio-sexual orientation than those who engage in non-consensual non-monogamy (Balzarini et al., 2020), in the sense that they see non-monogamy as a way to experience something new, explore sexual fantasies, and experience emotional and sexual variance (Haupert et al., 2017a; Rossman et al., 2019). Consensual non-monogamy has also been reported to positively affect the primary relationship functioning, especially for those with high socio-sexual background (Rodrigues et al., 2016).

Thus, we hypothesize that persons who have non-consensual non-monogamy have a lower relationship closeness to their primary partner than persons who do not have extradyadic experience and persons who have consensual non-monogamy. Furthermore, we hypothesize that persons who have consensual non-monogamy have more permissive attitudes toward sex and sexuality than persons who have non-consensual non-monogamy or no non-monogamy.

### Non-monogamy and STIs

Unprotected sexual intercourse with extradyadic partners may increase the potential risk of contracting STIs and passing on this infection to the primary partner. This will of course depend upon the type of sexual activities one engages in extradyadically, what knowledge one has of the extradyadic partner’s infection status, and the use of condoms for STI protection. The use of condoms among Europeans during sex with extradyadic partners is shown to be less widespread than desirable from a health perspective (Dubois-Arber & Spencer, 1998; Traeen et al., 2007). Most married or cohabiting persons are likely to believe they are their partner’s only sex partner. This perception of monogamy creates a feeling of security regarding unprotected sex (Beadnell et al., 2007; Magnus, 1998). Intense feelings of love and trust are associated with a tendency to perceive condoms as unnecessary (Pilkington et al., 1994). Insisting on condom use in a permanent relationship may raise suspicion, unpleasant questions, and reduce trust (Gavin, 2000). There are indications that non-consensual non-monogamy is associated with less condom use than consensual non-monogamy (Conley et al., 2012). Furthermore, many with non-consensual extradyadic experiences define themselves as monogamous individuals based on their emotions rather than sexual attachment to the primary partner (Swan & Thompson, 2016). As they perceive themselves as monogamous, they may feel more protected against sexual health risks, and use less condoms, than individuals who define themselves as being non-monogamous. On this background, we hypothesize that the use of condoms during non-monogamy is in general is low, and lower among those who have non-consensual non-monogamy than among those who have consensual activity. Accordingly, non-monogamy represents a risk for spreading STIs.

### Purpose

This study aims to describe the prevalence of non-monogamy in a web panel sample of the Norwegian population 2020. Based on the existing literature, we propose the following four hypotheses:

H1: Men have more experience of non-consensual non-monogamy than women, but there are no gender differences in consensual non-monogamy. The gender differences are less pronounced in younger than in older individuals.

H2: Individuals who identify as LGBT + have more experience of both non-consensual and consensual non-monogamy than individuals who identify as heterosexuals.

H3: Persons with consensual non-monogamy, or who have no non-monogamy experience, have a more intimate relationship with their primary partner and have more accepting attitudes toward sex and sexuality than persons who have non-consensual non-monogamy.

H4: The use of condoms during non-monogamy is low, particularly among those who have non-consensual non-monogamy. Accordingly, non-monogamy represents a risk for spreading STIs.

## Methods

### Participants

The results from this study are based on the responses from 4160 members of KANTAR’s Gallup Panel aged 18–89 years. The Gallup Panel is presented more in detail below. The recruitment of respondents was conducted in March-April 2020 by e-mail to a randomly selected sample of 11,685 Norwegians registered in Kantar’s Gallup Panel. Recruitment of participants continued until at least 4000 individuals had agreed to participate. The response rate in the present study was 35.6%, and 51% completed the survey on a mobile device. Results from this study are also published in other publications (Fischer & Traeen, 2021; Koletić et al., 2021; Træen et al., 2021; Træen & Fischer, 2021)

### Recruitment

The Gallup Panel consists of ∼46,000 members randomly recruited based on questionnaire surveys conducted by phone using probability samples. It is not possible to self-recruit for the Gallup Panel, and members of the Panel are supposed to be representative of the 98% of the population with access to the Internet (see http://www.medienorge.uib.no/english/), that is, Norway’s Internet population. The members are registered with a large set of social background variables (see Træen et al., 2021), which allows Kantar to select sub-samples according to different criterion variables (e.g. stratum, county, or community type), which makes it possible to draw specific target groups representing the desired sample. It is also possible to survey the same sample several times and to select sub-samples based on previous answers. Kantar operates with a small incentive to motivate participation, but not large enough to be the cause of participation in surveys.

KANTAR surveys follow the ethical guidelines developed for the market- and poll organization surveys (https://www.tnsglobal.com/press-release/we-are-strongly-committed-ethical-and-sustainable-practices). All members of the Gallup Panel are guaranteed safety and anonymity, and all participation in surveys is voluntary. Before this study, a pilot survey was conducted in a self-selected Facebook, and this pilot was approved by the internal ethical committee at the Department of Psychology.

### Survey questions

The questionnaire used in the study was developed by a group of researchers at the Department of Psychology, University of Oslo, based on the experience with the 2013 Norwegian Sex Study of 18–29 year-olds (Kvalem et al., 2014; Træen et al., 2016). The average time to complete the survey was 15 min.

### Sociodemographic characteristics of the sample

The sample consists of 52.4% men (*n* = 2181), 47.3% women (*n* = 1967), and 12 persons “other” (0.3%). In Kantar’s database, three of these participants were classified as men and nine as women. The age of the participants ranged from 18 to 89 years [mean age 46.5 years (SD 17.1 years); median age 44.0 years]. The men were somewhat older than the women [mean age_men_ 48.4 years (SD 17.1 years)/median age_men_ 48.0 years (range 18–87 years)/mean age_women_ 44.4 years (SD 16.8 years)/median age_women_ 41.0 years (range 18–89 years)]. With regards to sexual orientation, 93.5% reported to be heterosexual; 2.6% were homosexual/lesbian, 3.3% bisexual, and 0.6% asexual. Approximately six of 10 participants reported that they had no religious affiliation (59.5%), and 38.7% were Christians (mainly Protestants or Christians with no particular denomination). A total of 41.4% reported having a short university education, and 22.8% reported long university education. The majority of participants lived in urban areas (56.8%), and 16.3% lived in rural areas. The majority of participants lived with a partner (63.4%); one of four reported being unmarried (25.4%), 8.4% separated/divorced, and 2.8% widow/widower. Other sample characteristics are presented elsewhere (Traeen et al., 2021).

### The measures

Experiences of non-monogamy were measured by the following questions previously used in a survey of 18–29 year-olds in Norway in 2013 (Kvalem et al., 2014), and in the German Sex Survey 2019 (www.gesid.eu): “**Have you ever, while married or cohabiting, had sex with someone other than your primary partner?**” and “**Have you had other sex partners after you established your present permanent relationship, that is, extradyadic partners?**” The response categories for both questions were 1 = No; 2 = Yes, without my partner’s consent; and 3 = Yes, with my partner’s consent. Condom use was measured by the question “**Did you use contraception/protection the first time you had intercourse with your most recent extradyadic partner?**” The response categories were “No, none,” “Condom,” “Both condom and other contraception,” “Hormonal contraception (oral contraceptives, rings, patches, syringes, hormone IUD),” “IUD,” “Pessar,” “Spermicides,” “Interrupted intercourse or safe periods,” “Emergency contraception,” “Sterilization,” “Other protection,” “Uncertain/Don’t know,” and “Prefer not to answer.” For this study, the variable was recoded and dichotomized into 1 = “No condom use,” and 2 = “Condom use” (previous categories “Condom,” and “Both condom and other contraception”). To study the relationship with the most recent extradyadic partner we asked the questions “**What was your relationship with your most recent extradyadic partner?**” The response categories were “It was a one night stand,” “It was a longer term relationship,” “It was a service I paid for,” “Other, what?” and “Prefer not to answer;” “**What was the gender of your most recent extradyadic partner?**” The response categories were “Man,” “Woman,” “Non-binary/gender fluent,” and “Prefer not to answer;” “**How did you meet your most recent extradyadic partner?**” The response alternatives were “Through studies or work,” “Through acquaintances, friends or family,” “On a restaurant//bar/night club,” “Through a leisure time activity or on vacation,” “On social media (e.g. Facebook, Twitter, Instagram),” “Through a dating app or dating site,” “Other (please specify),” and “Prefer not to answer;” “**Was your most recent extradyadic partner in a permanent relationship?**” The response categories were “No,” “Yes,” “Don’t know,” and “Prefer not to answer,” and “**What kind of sex did you have the first time you had sex with your most recent extradyadic partner (tick all that apply)?**” The response alternatives were “Vaginal intercourse,” “Oral intercourse,” “Anal intercourse,” “Other,” and “Prefer not to answer.”

Other variables used in the statistical analyses in this paper were:

Age groups – The continuous age variable was recoded into four categories: 1 = 18–29 years, 2 = 30–44 years, 3 = 45–59 years, and 4 = 60+ years.

Sexual orientation – This was measured by the question **Do you currently regard yourself as: …,** The response alternatives were 1 = Homosexual/lesbian, 2 = Heterosexual, 3 = Bisexual/pansexual, 4 = Asexual, and 5 = Other. The variable was recoded into a dichotomous variable, 1 = LGBT + and 2 = heterosexual.

Emotional closeness to partner – This measure was adapted from Aron et al. (1992), and the German Sex Survey 2019 (www.gesid.eu). **These figures attempt to portray how close two persons may feel to each other. Choose the figure that best describes your relationship with your partner** (see illustration below). Intimacy was rated on a scale of 1 = low degree of intimacy to 7 = high degree of intimacy.

Relationship satisfaction – This was assessed by a question adapted from the German Sex Survey 2019 (www.gesid.eu): **All things considered, how satisfied are you with your current relationship?** Relationship satisfaction was rated on a scale of 1 = not satisfied at all to 7 = completely satisfied.

Attitudes toward sex and sexuality were taken from the 1996 Swedish Sex Study (Lewin et al., 2000), and were assessed by nine items following this introduction: “People are sexually stimulated by different things. Is this something you would be stimulated by, something you think you cannot do yourself but can accept that others can (for instance, your partner), or do you think it’s completely unacceptable?” The items were as follows: Being sexually stimulated by things, such as shoes or underwear/Being sexually stimulated by people of the same gender/Being sexually stimulated by ritual games connected to dominance and submission/Being sexually stimulated by dressing like the opposite gender/Being sexually stimulated by exposing oneself/Being sexually stimulated by using consensual dominance/submission/pain/Being sexually stimulated by spying on what others do sexually/Being sexually stimulated by violence/Being sexually stimulated by sending nude pictures. The response categories were 1= Can think of doing this myself; 2 = Cannot think of doing this myself, but would accept it in my partner; 3 = Cannot think of doing this myself, and would not accept it in my partner, but accept that others function that way; and 4 = Unacceptable.

### Statistical analyses

All data analyses were carried out using SPSS 25.0 for Windows. Contingency table analysis, and comparing means were used to study group differences. To the question of what kind of sex one had with the extradyadic partner, the participants could tick for as many response alternatives as necessary. Therefore, the cross-tabulation was carried out using multiple responses. To explore the differences in attitudes toward sex and sexuality, relationship satisfaction, and emotional closeness to the primary partner between three groups of participants (groups: monogamous, non-consensual non-monogamy, consensual non-monogamy), a discriminant analysis was performed. The variables were entered into the analysis using Wilk’s lambda (Klecka, 1980). A lambda of 1 occurs when the mean of the discriminant scores is the same in all groups and there is no between-group variability. Wilk’s lambda provides a test of the null hypothesis that the population means are equal. The larger the lambda value, the less discriminating power is present. The standardized canonical discriminant function coefficients show the relative association between the discriminating variables and discriminant functions.

## Results

### Prevalence of non-monogamy

Lifetime experience. To the question “Have you ever, while married or cohabiting, had sex with someone other than your primary partner?” 26.3% of the men and 17.8% of the women reported that they had ever engaged in non-consensual non-monogamy. The proportion who reported consensual non-monogamy was 3.1% of men and 2.6% of women. Gender differences within each age group were also studied (not shown in a Table). The proportion who reported consensual non-monogamy varied from 0% (in 18–29 year-old women) to 3.1% (in 30–44 year-old men). However, there was a statistically significant gender difference in the reporting of non-consensual non-monogamy in all age groups. In all age groups, men reported more experience of non-consensual non-monogamy than women did. The proportion who reported such experience increased from 6.0% of men and 2.6% of women aged 18–29 years (Chi-square 8.070, *p* = .018), to 23.5% of men and 13.5% of women aged 60+ years (Chi-square 11.079, *p* = .004).

Among individuals who identify as heterosexuals, 22.6% reported lifetime non-consensual non-monogamy, compared to 19.0% among individuals who identify as LGBT+, and 16.8% of individuals who identify as LGBT+ compared to 2.1% of individuals who identify as heterosexual reported consensual non-monogamy.

Experience in the current relationship. About twice as many men as women reported non-consensual non-monogamy in their current relationship (Table 1). The experience of non-consensual non-monogamy increased from 3.8% among participants younger than 30 years to 19.9% among those aged 60+ years. Lastly, consensual non-monogamy was reported by 16.2% of individuals who identify as LGBT+, and 1.2% of individuals who identify as heterosexuals, but there was no difference in the reporting of non-consensual non-monogamy.

**Table 1.** Non-monogamy in the current relationship, by selected social background variables (percentages).

Variables	N	No	Non-consensual	Consensual	*p*-Value
All	2595	85.1	13.1	1.8	
Gender					
Men	1403	80.5	17.4	2.1	.000
Women	1192	90.5	8.0	1.5	
Age groups					
18–29	367	95.4	3.8	0.8	.000
30–44	817	87.4	9.7	2.9	
45–59	661	83.7	14.7	1.7	
60+	750	78.9	19.9	1.2	
Sexual orientation					
LGBT+	99	69.7	14.1	16.2	.000
Heterosexual	2462	85.6	13.2	1.2	

*Note*. Tested for statistically significant differences between the groups by means of Chi-square test.

Table 2 shows the experience of non-monogamy by perceived closeness to one’s primary partner and relationship satisfaction. Participants who had engaged in non-consensual non-monogamy within their current relationship consistently reported lower levels of relationship satisfaction and feeling less close to their primary partner, compared to those who had not engaged in non-monogamy or engaged in consensual non-monogamy.

**Table 2.** Non-monogamy in the current relationship, by degree of intimacy with the primary partner.

Variables	No	Non-consensual	Consensual	*p*-Value
Degree of feeling close to the primary partner	5.0 (1.7)	4.2 (1.8)	5.0 (2.0)	.000
	*n* = 2156	*N* = 336	*n* = 46	
Relationship satisfaction	6.1 (1.1)	5.3 (1.5)	5.9 (1.4)	.000
	*n* = 2198	*N* = 338	*n* = 47	

Means (standard deviation).

*Note*. Tested for statistically significant differences between the groups by means of *F*-test.

The discriminant analysis to explore the differences between participants who had not engaged in non-monogamy during their current marriage or cohabitation (Table 3), those who had engaged in non-consensual, and consensual non-monogamy, attitudes toward sex and sexuality, relationship satisfaction, and closeness to the primary partner, resulted in two statistically significant discriminant functions (see Wilk’s lambda). The first discriminant function was dominated by the attitude toward sex and sexuality variables, and expresses how unaccepting or accepting individuals are toward a series of sexual expressions. For this reason, the first discriminant function was called “Attitudes toward sex and sexuality.” As can be seen from the magnitude of the standardized canonical discriminant coefficients, the second discriminant function was dominated by the variable on relationship satisfaction and perceived closeness to the primary partner. This discriminant function was called “Relationship intimacy.”

**Table 3.** Attitudes toward sex and sexuality and relationship to the primary partner according to three distinct groups of participants being monogamous, having experience of non-consensual or consensual non-monogamy in the current cohabiting relationship (discriminant analysis) (*n* = 1707).

	Attitudes toward sex and sexuality	Relationship intimacy
Being sexually stimulated by …	Pooled within group correlations between discriminating variables and standardized canonical discriminant functions	
… sending nude pictures	0.573*	−0.161
… using violence	0.539*	−0.513
… people of the same gender	0.534*	−0.345
… ritual games connected to dominance and submission	0.487*	−0.269
… spying on what others do sexually	0.481*	0.183
… exposing oneself	0.480*	0.104
… using consensual dominance/submission/pain	0.395*	−0.323
… things by things like shoes or underwear	0.270*	−0.165
… dressing like the opposite gender	0.130*	−0.102
Relationship satisfaction	0.457	0.611*
Emotional closeness to primary partner	0.411	0.466
	Canonical corr coeff.	
	0.307	0.196
	Wilk's lambda	
	0.871	0.962
	*p* < .001	*p* < .001
	Group centroids	
Monogamous	0.124	0.033
Non-consensual non-monogamy	−0.585	−0.363
Consensual non-monogamy	−1.617	1.109

*Note*. *Largest absolute correlation between each variable and any discriminant function.

**Figure 1.**
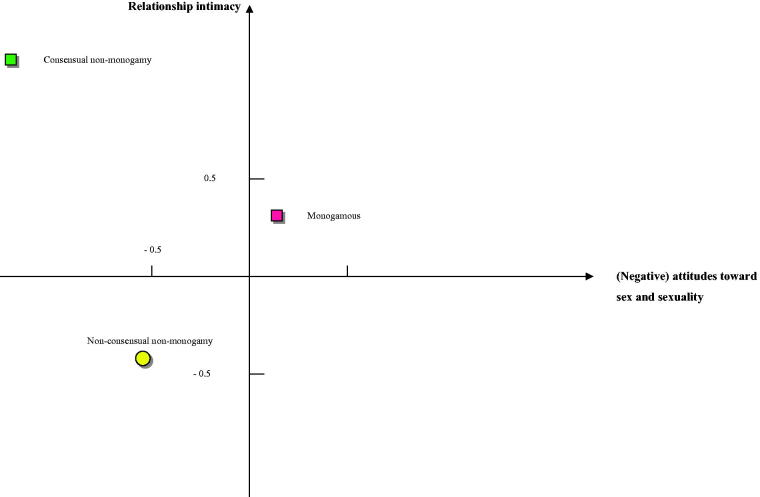
The placement of the discriminant groups in terms of group centroids along the two discriminant functions.

To focus more on group differences, the group centroids were studied. Group centroids are the mean discriminant scores for each group on the respective functions. The centroids summarize the group locations in the space defined by the discriminant functions. This is visualized in Figure 1, where the group centroids are plotted on a graph defined by the two discriminant functions.

Monogamous participants in the current relationship grouped in the direction of the positive pole on the “Relationship intimacy” function, and close to the point of intersection between the two functions on the “Attitudes toward sex and sexuality” function. This indicates that compared to those with extradyadic experience, participants who had no extradyadic experience were characterized by relatively higher relationship intimacy and neutral attitudes toward sex and sexuality. Participants with non-consensual extradyadic experience grouped somewhat to the negative pole on the “Relationship intimacy” function, and somewhat to the negative pole on the “Attitude toward sex and sexuality” function. The group with consensual non-monogamy grouped close to the positive pole on the “Relationship intimacy” functions and close to the negative pole on the “Attitude toward sex and sexuality” function. Accordingly, compared to participants with no or non-consensual non-monogamy in their current relationship, participants with consensual non-monogamy were very highly related to relationship intimacy and had very positive attitudes toward trying out different sexual expressions.

### The most recent non-monogamy

Of the participants with extradyadic sexual experience in their current relationship (Table 4), the majority reported that their most recent extradyadic partner was a one-night stand/casual relationship, one of three said it was a long-term relationship, and a minority had paid for the sex or reported “other.” About one of two women and one of three men reported having met the extradyadic partner through studies or work. The second most frequently reported means of meeting the extradyadic partner was through acquaintances, friends, or family, followed by restaurants/bars, leisure time activities/vacations, and dating apps. A minority of men and women reported that their most recent extradyadic partner had the same gender as themselves. There was no statistically significant gender difference in reporting.

**Table 4.** Circumstances around the most recent non-monogamous incident, by gender (percentages).

	All	Men	Women	*p*-Value
Relationship to the most recent extradyadic partner				
It was a one night stand	57.4	59.5	52.4	ns
It was a longer term relationship	33.5	32.4	36.3	
It was a service I paid for	3.3	3.9	1.6	
Other	5.8	4.2	9.7	
*N*	430	306	124	
Gender of the most recent extradyadic partner				
Same-sex partner		13.4	8.9	ns
Other-sex partner		86.6	91.1	
* N*		306	124	
Where the most recent extradyadic partner was met				
Through studies or work	38.5	34.4	49.2	ns
Through acquaintances, friends or family	16.8	16.7	16.9	
On a restaurant//bar/night club	12.5	14.4	7.6	
Through a leisure time activity or on vacation	12.1	12.5	11.0	
On social media (e.g. Facebook, Twitter, Instagram)	4.3	4.9	2.5	
Through a dating app or dating site	9.2	9.5	8.5	
Other (please specify)	6.6	7.5	4.2	
*N*	423	305	118	
Extradyadic partner in a relationship				
No	46.8	48.5	42.3	**
Yes	41.0	36.6	52.0	
Don’t know	12.3	14.9	5.7	
* N*	432	309	123	

ns: not statistically significant.

*Note*. Tested for statistically significant gender differences by means of the Chi-square test.

**p < .01.

To the question of whether or not the extradyadic partner was in a permanent relationship, more women than men said *yes* (Chi-square = 11.937, *p =* .003).

Regarding condom use (Table 5), about three times as many men aged 18–29 years as men aged 60+ years claimed they used condoms the first time they had sex with their most recent extradyadic partner. At this time, about one in five men individuals who identify as heterosexual and one in two men who identify as LGBT + reported condom use. Women generally reported less condom use, and there was no statistically significant difference in the reporting by age groups or sexual orientation. About three of ten of those with consensual extradyadic experience reported condom use, compared to about one of five those without consensual extradyadic experience. However, this difference was not statistically significant (*p* = .258). The three most commonly reported reasons for not having used condoms among men were “I felt we were both healthy,” “There was no condom available,” and “Sex with condoms is less sensual.” Likewise, the three most commonly reported reasons among women were “I felt we were both healthy,” “Other reasons,” and “There was no condom available.”

**Table 5.** Condom use during the first sexual intercourse with the most recent extradyadic partner (percent).

	Men	Women
Age group		
18–29 years	43.5***	11.1^ns^
30–44 years	36.8	25.0
45–59 years	22.5	23.3
60+ years	13.8	11.6
* N*	311	126
Sexual orientation		
LGBT+	47.1***	8.3^ns^
Heterosexual	21.5	19.5
* N*	309	125

ns: not statistically significant.

*Note*. Tested for statistically significant gender differences by means of the Chi-square test.

***p < .001.

The type of sex during the first sexual intercourse with the most recent extradyadic partner was examined. In both men and women, the majority reported vaginal sex, followed by oral sex, anal sex, and “other” sexual practices. Table 6 shows the type of sex by age group and sexual orientation for men and women. In both sexes, the prevalence of vaginal sex increased with age, whereas oral sex and anal sex decreased with increasing age. Heterosexuals of both genders reported mainly vaginal sex, followed by oral sex. Men who identify as LGBT+ reported mainly anal sex and oral sex, and women who identify as LGBT+ reported mainly oral sex and vaginal sex.

**Table 6.** Type of sex during the first sexual intercourse with the most recent extradyadic partner (multiple response, percent).

	Men				Women			
	Vaginal sex	Oral sex	Anal sex	Other	Vaginal sex	Oral sex	Anal sex	Other
Age group								
18–29 years	43.5	47.8	43.5	8.7	37.5	50.0	12.5	12.5
30–44 years	64.4	49.3	16.4	6.8	72.7	38.6	4.5	4.5
45–59 years	80.7	46.6	6.8	11.4	79.3	37.9	3.4	3.4
60+ years	88.4	19.8	2.5	5.0	82.9	9.8	3.4	9.8
*N*	235	112	31	23	92	36	4	8
Sexual orientation								
LGBT+	15.2	48.5	54.5	15.2	36.4	54.5	9.1	9.1
Heterosexual	84.9	35.1	4.8	6.6	80.0	26.4	2.7	6.4
*N*	235	111	31	23	92	35	4	8

*Note*. Not tested for statistically significant differences as multiple response is applied.

## Discussion

To sum up the findings from the present study, approximately one of four men (26%) and one of five women (18%) had a lifetime experience of non-consensual non-monogamy, and about 3% of both genders had the experience of consensual non-monogamy. In their current marriage or cohabitation, less than one of five men and less than one of 10 women engaged in non-consensual non-monogamy, and about 2% engaged in consensual non-monogamy. Comparing these estimates to previous findings is difficult due to different definitions and operationalizations of “extradyadic sex” (Blow & Hartnett, 2005b). Even so, we would claim that the findings from this study largely support the findings of previous Norwegian studies (Træen et al., 2007; Træen & Stigum, 1998) as well as research from other countries (Hackathorn & Ashdown, 2021; Haupert et al., 2017a, 2017b). In a recent update, studies estimate that around 25% of married men and 15% of married women have had extradyadic sexual relations (Hackathorn & Ashdown, 2021). In 2002, 29% of Norwegian men aged 18–49 years and 23% of women reported lifetime experience of non-monogamy, and 16% of men and 11% of women engaged in non-monogamy in their current marriage (Træen et al., 2007). Although not directly comparable with this study, the findings suggest there has been little overall change from the 1990s to 2020 in the prevalence of non-monogamy in the Norwegian population.

We first hypothesized that men would have more experience of non-consensual non-monogamy than women, but that there would be no gender differences in consensual non-monogamy. We also hypothesized that gender differences would be less pronounced in younger than in older individuals. Hypothesis 1 was verified. For both men and women, the prevalence of non-consensual extradyadic sexual experience increased statistically significantly with the increasing age group. This finding has also been reported in other studies (Kontula & Haavio-Mannila, 1995; Lewin et al., 2000). More of the older participants reported such experience, as they have had the time to accumulate this experience. The increase in non-consensual non-monogamy with age can also be seen as a function of how long the primary relationship has lasted, and may therefore not be a true age development effect (Liu, 2000; Træen & Stigum, 1998). Based on previous research, gender differences in the prevalence of extradyadic experiences were expected (Hackathorn & Ashdown, 2021; Hubert et al., 1998; Lewin et al., 2000; Træen et al., 2007). However, when we examined the circumstances around the most recent extradyadic relationship, few gender differences were detected in this study. This indicates that women and men having non-monogamy may have become more similar. Even so, it is worth noticing that women more often than men reported having met their most recent extradyadic partner through studies or work. This may have had an impact on the perception of quality of the extradyadic relationship, in the sense that this may make the individual more emotionally connected to the extradyadic partner compared to a previously unknown partner (Blow & Hartnett, 2005a; Buss & Shackelford, 1997; Daneback et al., 2009; Træen, 1998; Træen et al., 2007).

The second hypothesis was that individuals who identify as LGBT + will have more experience of both non-consensual and consensual non-monogamy than individuals who identify as heterosexuals. We found that sexual orientation was associated with consensual non-monogamy, and the hypothesis was therefore only partly confirmed. Regarding consensual and non-consensual non-monogamy jointly, this corresponds to previous research (Blow & Hartnett, 2005a). Kurdek (1991b) found that a higher percentage of same-sex couples than mixed-sex couples had extradyadic sexual partners. As suggested by Kontula and Haavio-Mannila (1995), the gay subculture may not share the heterosexual *love script* and does not regard non-monogamy as infidelity. Within such a permissive subculture, pleasure theory (Abramson & Pinkerton, 2002), may provide another explanation for the observed differences in consensual non-monogamy, and the search for sexual pleasure is seen as the main drive for seeking sex with extradyadic partners (Matsick et al., 2021). Furthermore, since Kurdek’s studies, HIV-prevention medication has become available for men who have sex with men, and this may explain why men who identify as LGBT + engage in more non-monogamy, as well as more anal sex than men who identify as heterosexuals, and why men who identify as LGBT + fail to use condoms in potentially risky sexual situations, such as unprotected anal sex with extradyadic partners.

H3: Persons with consensual non-monogamy, or who have no non-monogamy experience, have a more intimate relationship with their primary partner and have more accepting attitudes toward sex and sexuality than persons who have non-consensual non-monogamy.

The third hypothesis was that persons with consensual non-monogamy, or who have no non-monogamy experience, have a more intimate relationship with their primary partner and have more accepting attitudes toward sex and sexuality than persons who have non-consensual non-monogamy. This hypothesis was confirmed. This also corresponds to previous findings (Blow & Hartnett, 2005a). The plotting of the group centroids gives an image of distinct systems of attitudes toward sex and sexuality and relationship intimacy between participants with no, non-consensual, and consensual extradyadic experiences. The mapping of groups shows the distance between these groups. Of particular interest is how the consenting participants are particularly different from the other groups regarding attitudes toward sex and sexuality and relationship intimacy, and how non-consenting participants are positioned in different compartments on the relationship intimacy axis. Participants who had no extradyadic experience were characterized by relatively higher relationship intimacy and neutral attitudes toward sex and sexuality. Participants with non-consensual extradyadic experience had comparatively low relationship intimacy but had more positive attitudes toward sex and sexuality. Participants with consensual extradyadic experience were high in both relationship intimacy and positive attitudes toward sex and sexuality. This finding supports Balzarini et al. (2020) findings that swingers have the most permissive sexual attitudes and are the most unrestricted sexually, whereas strictly monogamous individuals were the opposite.

H4: The use of condoms during non-monogamy is low, particularly among those who have non-consensual non-monogamy. Accordingly, non-monogamy represents a risk for spreading STIs.

The fourth hypothesis was partly confirmed. Non-monogamy was related to low condom use, but not we found no difference in condom use between people with non-consensual and consensual non-monogamy. About one of two women, and one of three men, reported having met the extradyadic partner through studies or work. This indicates that the place of work/studying is still an important arena to meet the extradyadic partner (Kontula & Haavio-Mannila, 1995; Træen et al., 2007). More importantly, this also indicates that those who engage in non-monogamy are likely to have prior knowledge of each other; this may explain the relatively low use of condoms, particularly among middle-aged and older individuals, and explain why the non-condom users trust they are healthy. This is by Swan and Thompson (2016) finding that individuals in self-defined monogamous relationships may feel more protected against sexual health risks and use less condom than individuals who define their primary relationship as non-monogamous.

Two decades ago, 29% of people living in the Norwegian capital, Oslo, used condoms during their most recent intercourse with an extradyadic partner (Træen et al., 2002). Our study reveals that at the first occasion of sex with the extradyadic partner, condom use was low, particularly among heterosexuals. Men who identify as LGBT + were much better condom users than men who identify as heterosexual. Only one of five men who identify as heterosexual and nearly every other man who identifies as LGBT + reported condom use, and penetrative sexual activity was frequent. This raises the question if, and how, safe sex is negotiated in extradyadic settings (Swan & Thompson, 2016). Denes and Speer (2018), analyzed 819 ads that partnered individuals seeking extra-dyadic sexual relationships on Craigslist, to explore how safe sex was negotiated. They found that partnered men seeking sexual activity with other men posted the most ads and had the largest percentages for several keywords related to safe sex, whereas this was not as common in heterosexual constellations. In the wake of the AIDS epidemic, it may have become more accepted for men who identify as LGBT + to negotiate safe sex and use condoms.

Either way, the low condom use makes non-monogamy a risk situation for STIs. As every other participant reported that the extradyadic partner was in a permanent relationship or this was uncertain, many of those engaging in non-monogamy risk being infected with STIs. Studies have shown that most often, heterosexuals use condoms to protect against unwanted pregnancy and not primarily against STIs (Træen & Hovland, 1998). In men who identify as LGBT+, the introduction of hiv-prevention medication may explain the low condom use.

### Limitations

Previous Norwegian sexual behavior surveys have shown a drop in response rates from 63% in 1987 to 21% in 2008 (Træen & Stigum, 2010; Træen et al., 2007). In this respect, the response rate of 35.6% in this study can be said to be fairly good. Even so, the response rate may pose problems regarding generalizing the results to the general population of Norway. Stigum (1997) studied dropouts in the sexual behavior study 1992 and concluded that the dropouts were random rather than systematic. Likewise, Træen et al. (2003) concluded that dropouts in the 1997 and 2002 surveys were not likely to be biased. When we compare the results from this study to the previous Norwegian sex surveys, we believe that dropouts in this survey are random rather than systematic. However, compared to the general population our sample may be somewhat biased about the proportion of participants who had more than 14 years of education (Træen et al., 2021).

Different operationalizations and definitions throughout the literature make comparison with other studies difficult (Blow & Hartnett, 2005b). We did not define for the respondents what was meant by “sex partners.” However, by asking what kind of sex they had with their most recent extradyadic partner, we could have a fuller understanding of that. It should also be noted that this study focused only on sexual non-consensual non-monogamy as opposed to other forms that exist (e.g. online). Lastly, the cross-sectional nature of this study makes it impossible to conclude cause and effect.

## Conclusions

Based on the results of this study, it can be concluded that the monogamy norm is still strong in Norwegian society. The majority of the population is monogamous and does not report engaging in non-monogamy. Of those who have extradyadic sex, the majority engages in non-consensual non-monogamy, but about 3% of the population engages in consensual non-monogamy and thus breaks with the norms related to monogamy (Træen et al., 2007). The monogamy norm is challenged by this small group of individuals who have their partner’s consent to have sex with extradyadic partners.

There are important lessons to be learned from this study for future research. First, it is necessary to define for respondents what is meant by the different terms and concepts introduced (Blow & Hartnett, 2005b). It should be explicitly defined what is meant by for instance concepts, such as “extradyadic partner,” “extradyadic sexual activity,” or “non-monogamy.” Furthermore, future research should avoid asking questions about “infidelity,” as this concept is morally loaded, and instead focus on non-consensual and consensual non-monogamy. Also, more studies are needed to explore the social reality of people who engage in consensual non-monogamy as this is something we know little of to the present. For example, to what extent do these couples have an explicit sexual agreement about non-monogamy? A previous study indicates that many mixed-sex couples do have some kind of sexual agreement (Lee & Mitchell, 2017), but that this is more common in consensual non-monogamy relationships (Mogilski et al., 2017).

Lastly, the results of this study illustrate the complexity underlying research on non-monogamy. Future research should focus more on understanding the factors behind the low rate of condom use during such events. In this context, a qualitative research approach may be useful to understand why condom use is neglected, and whether or not there is a difference between those who engage in consensual and non-consensual nonmonogamy in this respect. This may be of importance also for health promotion and getting the right message out to the specific target group.
